# Evidence of Subterranean Termite Feeding Deterrent Produced by Brown Rot Fungus *Fibroporia radiculosa* (Peck) Parmasto 1968 (Polyporales, Fomitopsidaceae)

**DOI:** 10.3390/insects7030041

**Published:** 2016-08-18

**Authors:** Nadia Nuraniya Kamaluddin, Akiko Nakagawa-Izumi, Shota Nishizawa, Ayuko Fukunaga, Shuichi Doi, Tsuyoshi Yoshimura, Sakae Horisawa

**Affiliations:** 1 Graduate School of Life and Environmental Sciences, University of Tsukuba, Tsukuba 305-0872, Japan; nadia.kamaluddin@gmail.com(N.N.K.); nishizawa@d-m-b.co.jp, (S.N.); s1336502@u.tsukuba.ac.jp (A.F.); sd9642@yahoo.co.jp (S.D.); 2 Research Institute for Sustainable Humanosphere, Kyoto University, Uji, Kyoto 611-0011, Japan; tsuyoshi@rish.kyoto-u.ac.jp; 3 School of Environmental Science and Engineering, Kochi University of Technology, Kami, Kochi 782-8502, Japan; horisawa.sakae@kochi-tech.ac.jp

**Keywords:** decay fungus, *Reticulitermes speratus*, no-choice feeding, wood

## Abstract

We found that decayed wood stakes with no termite damage collected from a termite-infested field exhibited a deterrent effect against the termite *Reticulitermes speratus*, Kolbe, 1885. The effect was observed to be lost or reduced by drying. After identification, it was found that the decayed stakes were infected by brown rot fungus *Fibroporia radiculosa* (Peck) Parmasto, 1968. In a no-choice feeding test, wood blocks decayed by this fungus under laboratory condition deterred *R. speratus* feeding and *n*-hexane extract from the decayed stake and blocks induced termite mortality. These data provided an insight into the interaction between wood-rot fungi and wood-feeding termites.

## 1. Introduction

Wood-feeding termites and wood-decaying fungi both degrade and digest wood for energy, suggesting an interaction between these two groups. Studies on the interactions between termites and wood-decaying fungi have mainly focused on investigating the preference of termites with respect to different species of decayed wood. Pine wood blocks infected with the brown rot fungus *Gloeophyllum trabeum* (Pers.) (Murrill, 1908) induce *Reticulitermes flavipes* (Kollar, 1837), *R. virginicus* (Banks, 1907), and *Nasutitermes columbicus* (Holmgren, 1910) to form aggregations [1]. Matsumura et al. [2] identified the attractant in wood decayed by *G. trabeum* as (*Z,Z,E*)-3,6,8-dodecatrien-1-ol. In addition, Cornelius et al. [3] showed that sawdust infected with *Marasmiellus troyanus* (Murrill) (Dennis, 1970) is preferred by *Coptotermes formosanus* (Shiraki, 1905).

Several studies have shown the termite feeding deterrence phenomenon in relation to wood decayed by fungi. Amburgey and Beal [4] showed that white rot-decayed southern pine stakes were not a preferred food substance for *Reticulitermes flavipes* (Kolar). Furthermore, stakes of *Pinus elliotti* (Engelm), *P. taeda* L., and *P. palustris* sapwood infected with a white rot fungus were not damaged by termites, but damage was caused by light feeding. Grace et al. [5] demonstrated that secondary metabolites released by the brown-rot fungus *G. trabeum* deterred *C. formosanus* feeding on filter paper.

Fungi and insects use chemosensory systems to communicate and interact with the environment. Sharing of living space by these two organisms has led to the evolution of a wide range of beneficial as well as adverse interactions [6]. Compounds associated with fungi-insect communication exist in various forms, with fungal secondary metabolites occurring as kairomones, allomones [7], and siderophores [8]. In termite-infested areas, termites tend to avoid feeding on the decayed parts of wood, suggesting that they are prevented from approaching or feeding on wood by decay-related chemical substances secreted by the fungi. Siderophores, at high concentrations, might act as chemosensory signals for various substrates, and are indicators of excessive degradation and, hence, of the absence of nutritional content. The secretion of feeding deterrents is also beneficial to fungi because they eliminate other competitors for the same cellulosic resource [5].

In this study, we identified the fungus that causes termite feeding deterrence on field decayed stakes. Specifically, we examined the feeding deterrence phenomenon of decayed stakes from the field, and evaluated this phenomenon under laboratory conditions. Furthermore, extracts of decayed stakes and wood were applied to paper disks to investigate any deterrent effect.

## 2. Materials and Methods

### 2.1. Termites

*R. speratus* (Kolbe, 1885) colonies were collected from the Living Sphere Simulation Field, Research Institute for Sustainable Humanosphere (RISH), Kyoto University (Kagoshima Prefecture, Japan). Multiple colonies obtained from this location were used in all of the tests, except for the feeding test of *n*-hexane extract from wood decayed under laboratory conditions which were collected from a pine forest in Oarai City (Ibaraki Prefecture, Japan). All of the termite colonies were housed in plastic containers in a temperature and moisture controlled laboratory until use. Different colonies were used for each test; none of the colonies were used twice.

### 2.2. Decayed Stakes

Soil-buried Japanese red pine (*Pinus densiflora* sp.) sapwood stakes (30 mm × 30 mm × 300 mm) were collected from the Living Sphere Simulation Field, RISH, Kyoto University, in October 2008. Two kinds of stakes were collected: decayed and sound. Decayed stakes were partly covered with hyphae and had clear signs of termite feeding (e.g., tunnels, presence of excrement, or attached living termites); sound stakes did not show signs of fungal decay or termite feeding. These stakes were placed in separate polyethylene bags and stored at ambient temperature to prevent drying.

### 2.3. Preliminary Observation

A decayed stake was selected from the collected Japanese red pine stakes. Three specimens (approximately 10 mm × 27 mm × 15 mm) were prepared from the decayed section and another three were prepared from the sound section. These specimens were exposed to 1000 *R. speratus* workers inside a plastic box (size: 200 mm × 270 mm × 150 mm) containing damp sand and were kept at room temperature for 14 days. Termite aggregations were observed every 24 h for 10 consecutive days.

### 2.4. Two-Choice Feeding Test of Air and Oven Dried Field Stakes

Three decayed stakes numbered 1–3 (30 mm × 30 mm × 300 mm) were trimmed into 15 mm × 15 mm × 5 mm specimens. From the decayed part, six specimens were prepared and divided into two groups, air-dried and oven-dried. Air-dried specimens were prepared by drying at room temperature for 48 h and oven-dried specimens were prepared by drying at 60 °C for 48 h. Sound specimens were prepared by cutting the not-decayed part of each stake and dried at 60 °C for 48 h before weighing.

The dried specimens were compared with sound specimens in separate two-choice feeding tests. In each test, air-dried and sound specimens or oven-dried and sound specimens were exposed to 1000 *R. speratus* workers from a single colony inside a plastic box (200 mm × 270 mm × 150 mm) containing damp sand, and kept at room temperature for 14 days. After the exposure, wood specimens were removed, cleared of any attached debris, oven-dried at 60 °C for 48 h, and weighed. The mass loss of the wood specimens was calculated from the differences in dry weight before and after exposure. The weights of the air-dried and oven-dried specimens before exposure were determined from the dry weights and moisture contents of specimens cut from an adjacent section of the stake.

### 2.5. Preparation of Wood Specimens from the Stakes and No-Choice Feeding Test

Three decayed stakes (numbered as 4–6) were selected. From each stake, five non-dried small specimens (approximately 5 mm × 5 mm × 5 mm) were trimmed, weighed, and immediately introduced to the no-choice feeding test using *R. speratus*. Control specimens were prepared from a different stake without fungal and termite damage. Both decayed and sound specimens were subjected to a no-choice feeding test.

No-choice feeding tests were conducted as follows. Decayed and control specimens were placed in individual plastic saucers on top of 15-mm-thick hard plaster (New Plastone Dental Stone, GC Corp., Tokyo, Japan) covered with wet sand in a 100-mL plastic cup. The bottom of each cup was perforated (the diameter of the pore was 10 mm) to supply moisture through the plaster layer that was in contact with a damp paper pad placed at the bottom of the test chamber. One hundred *R. speratus* workers from one colony were introduced to each cup. Test chambers were maintained inside an incubator at 27 °C. After the exposure, wood specimens were removed, cleared of any attached debris, oven-dried at 60 °C for 48 h, and weighed. Mass loss calculation was done in the same manner as described in the previous section.

### 2.6. Feeding Test Using Paper Disks Immersed with N-Hexane Extract from a Decayed Stake

Decayed stakes were dried using a freeze-drier and ground with a mortar and pestle. Five grams of the ground stake was extracted using 100 mL *n*-hexane at room temperature for 24 h. The extract was concentrated to 10 mL in a rotary evaporator at 10 °C. The concentration of the extract obtained by this procedure was 8%. The *n*-hexane extract of a sound stake was prepared in the same manner.

Paper disks (8 mm in diameter, thick type; ADVANTEC TOYO, Tokyo, Japan) were treated with the concentrated extract and kept at room temperature to remove the solvent. No-choice feeding tests were conducted using disks following the method described in previous section, with the exception that 50 workers were introduced per cup. This test was conducted using a single termite colony originating from the RISH field. Paper disks immersed in the solvent alone served as controls. Five replicates were tested for 10 days at 27 °C. After the test, the paper disks were removed, cleared of any attached debris, oven-dried at 60 °C for 48 h, and weighed. The mass loss of the disks was calculated from the difference in dry weight before and after the exposure.

### 2.7. Identification of Wood-Decaying Fungi Isolated from Decayed Stakes

Small pieces of decayed sections were sampled to isolate wood-decay fungi from the stakes by placement on potato dextrose agar (PDA) medium plates containing 100 ppm benlate and 50 ppm tetracycline hydrochloride to prevent mold and bacterial growth. The isolation procedure was conducted at 20 °C.

Three fungi were identified using the nucleotide sequence of the ribosomal DNA ITS region. The fungal hyphae were scraped from the colony on the PDA plate, placed in a microtube with zirconia beads, and homogenized with a beater at 25 Hz for 2 min. DNA extraction from the hyphae was performed by ISOPLANT II (Nippon Gene, Toyama, Japan). The obtained DNA was dissolved in a sterile Tris EDTA (TE) buffer.

The polymerase chain reaction (PCR) mixture was prepared using TaKaRa Ex Taq (TaKaRa Bio, Shiga, Japan), and primers for ITS1 (5’-TCCGTAGGTGAACCTGCGG-3’) and ITS4 (5’-TCCTCCGCTTATTGATATGC) were added. PCR was performed with a 2720 Thermal Cycler (Applied Biosystems, Foster City, CA, USA) with the following parameters: initial denaturation for 3 min at 94 °C; 25 cycles of denaturation for 15 s at 94 °C, annealing for 30 s at 55 °C, and extension for 30 s at 72 °C; and a final extension for 5 min at 72 °C. The PCR products was ligated into the pGEM-T Vector (Promega, Madison, WI, USA) and transformed into *Escherichia coli* JM 109 (TaKaRa Bio). Transformed strains were cultivated for 1 day, and their plasmids were purified using a Miniprep DNA Purification Kit (TaKaRa Bio). Nucleotide sequences were determined by a commercial sequencing service (Solgent, Daejeon, Korea). The ITS region sequences were analyzed using BLAST to find the most similar database sequences available. 

### 2.8. Preparation of Decayed Wood and No-Choice Feeding Test under Laboratory Conditions

Potato Dextrose Agar (PDA) medium, 1/3 diluted, was prepared by adding 1.3 g of PDA powder (Nissui Pharmaceuticals, Tokyo, Japan) and 2.6 g of agar (Nacalai Tesque, Kyoto, Japan) to 100 mL tap water to obtain medium of suitable hardness. The medium was poured into a 500-mL screw-capped bottle and sterilized by autoclaving at 121 °C for 15 min. Sterilized medium was inoculated with the isolated fungus *F. radiculosa* and incubated at 27 °C for 10 days. Block samples of Japanese red pine sapwood (20 mm × 20 mm × 5 mm) were oven-dried at 60 °C for 48 h, weighed, and sterilized with ethylene oxide gas. Three wood block samples were placed on a hyphal mat grown in a bottle and incubated at 27 °C. Wood blocks were aseptically transferred, after 8 weeks, to new medium to maintain suitable decay conditions. After 16 weeks of incubation, the surface hyphae was carefully removed from the wood block samples. All decayed wood blocks were kept in polyethylene bags and stored in a freezer until use.

For the no-choice feeding test, 20 mm × 20 mm × 5 mm blocks were trimmed into smaller specimens (5 mm × 5 mm × 5 mm) and divided into three groups: non-dried, air-dried (dried at room temperature for 48 h), and oven-dried specimens (dried at 60 °C for 48 h). Specimens were placed individually inside a plastic cup (identical to cup in Section 2.5) and tested against 100 *R. speratus* workers. The test was conducted under the same conditions as the no-choice feeding test for decayed stakes mentioned in Section 2.5, and ten sound Japanese red pine sapwood blocks (5 mm × 5 mm × 5 mm) were used as controls. Five replicates were conducted for each group and a colony from the RISH field was used in this test. The no choice feeding test using non-dried and control specimens was conducted first, followed by the no choice feeding test using air-dried, oven-dried and control specimens. The residual portion of the trimmed decayed wood blocks was oven-dried at 60 °C for 48 h and weighed to determine moisture content and mass loss by decay.

### 2.9. Preparation of N-Hexane Extracts from Wood Decayed by F. radiculosa

Three Japanese red pine sapwood sections (20 mm × 20 mm × 5 mm) were oven-dried at 60 °C for 48 h, weighed, sterilized with ethylene oxide gas, and exposed to the hyphal mat of *F. radiculosa* previously grown in a 500-mL bottle on PDA medium. After incubation at 27 °C for 2 months, wood blocks were removed, and the surface hyphae carefully removed. The wood blocks were dried in a freeze-drier and ground with a mortar and pestle. Five grams of the ground wood was extracted with 100 mL *n*-hexane at room temperature for 24 h. The extract was filtered using phase separator paper (ADVANTEC TOYO), and concentrated using a rotary evaporator at room temperature to approximately 5 mL, when a deep yellow color appeared.

### 2.10. Feeding Test Using Paper Disks Immersed in N-Hexane Extract from Laboratory-Decayed Wood

Paper disks (8 mm in diameter, thick type; ADVANTEC TOYO, Tokyo, Japan) were treated with the extract obtained from laboratory-decayed wood. A sound wood extract (8%), was prepared under conditions similar to the decayed wood extract. This experiment was conducted to validate that feeding deterrence was not correlated with the sound wood extract. Extract-treated paper disks were kept at room temperature to remove the solvent used in the extraction process. No-choice feeding tests were conducted with the paper disks using 50 termite workers that were introduced into each cup. A single colony from Oarai City, Ibaraki Prefecture, was used in this test. Paper disks immersed in the solvent served as controls. Three replicates were tested for 10 days at 27 °C. After the test, the paper disks were removed, cleared of any attached debris, oven-dried at 60 °C for 48 h, and weighed. The mass losses of the disks were calculated from the difference in dry weight before and after exposure.

### 2.11. Statistical Analysis

The data from the two-choice feeding test between air-dried, oven dried, and sound stake specimens (Figure 3) were analyzed using analysis of variance (ANOVA). Kruskal-Wallis H tests were run to determine differences between termite feeding of decayed stakes and wood under laboratory conditions (Figure 4 and Figure 6) in no-choice feeding tests and feeding and mortality from decayed stakes and wood extracts (Figure 5 and Figure 7). Test significance levels were set to α = 0.05 and computed using IBM SPSS Statistics version 2.2.

## 3. Results

### 3.1. Feeding of the Stakes

**Figure 1:**
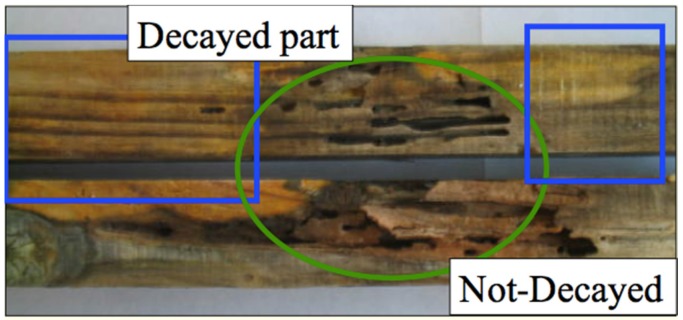
Picture showing the avoidance of the decayed part of the field stakes by termites (**blue boxes**) and feeding on the not-decayed parts (**green circle**).

It was observed that termite damage was not detected on the fungus-decayed part of field stakes collected at the RISH field in Kagoshima Prefecture (Figure 1). Decayed parts were defined by the presence of surface hyphae or mycelium and visibly lighter brown color (blue squares) whereas in the not-decayed part, no hyphae or mycelium were detected and the area was darker (green circle). In the preliminary feeding test using one of the field stakes, termite feeding was focused on wood specimens prepared from the decayed part in the first 3 days of the test.

**Figure 2:**
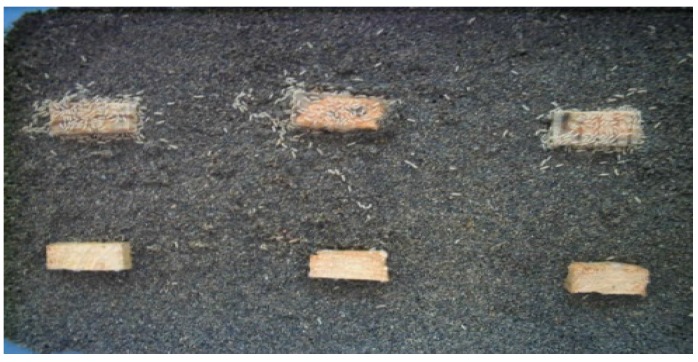
Termite aggregations on sound (**upper blocks**) and decayed (**lower blocks**) specimens of the same stake after the first 3 days of the choice feeding test. This aggregation pattern persisted until the end of the 14-day exposure period.

During the initial stage of the test, termites aggregated around the specimens prepared from the decayed stakes (Figure 2); however, after three days of exposure, most of the termites aggregated around the specimens prepared from the sound stake. This preference persisted until the end of the 14-day exposure period.

No mass loss difference was detected in two-choice feeding tests between oven-dried specimens and sound specimens, as well as between air-dried specimens and sound specimens.

Three decayed stakes obtained from the same field (labeled as No. 4, 5, and 6) were subjected to the no-choice feeding test. Significant mass loss difference was detected at α = 0.05, X^2^(3) = 12.198, *p* = 0.007 (Kruskal-Wallis H test).

### 3.2. Effects of the N-Hexane Extract from the Decayed Stake on Termite Feeding

The mass loss of the paper disks immersed in *n*-hexane extract from decayed and sound stakes, and termite mortality are shown in Figure 5. Kruskal-Wallis H tests were conducted to determine differences among filter paper mass loss and mortality. Distribution of termite feeding was similar for all groups; non-dried decayed wood extract feeding was lower than that of the solvent and sound wood extract, and the differences were not statistically significant at α = 0.05, X^2^(2) = 5.600, *p* = 0.061). Significant differences between groups were detected in termite mortality (X^2^(3) = 9.630, *p* = 0.022).

### 3.3. Identification of the Isolated Basidiomycete from the Stakes

Fungi isolated from different stakes were categorized according to morphological characteristics such as white to creamy-white fungal mat and light yellow color over the inoculum and surrounding zone. The fungi was identified as the brown rot basidiomycete *Fibroporia radiculosa* (Peck) Parmasto 1968. This result was supported by the description in Lombard and Gilbertson [9].

### 3.4. Feeding Test of Specimens Decayed by F. radiculosa under Laboratory Conditions

The percent mass loss of wood specimens prepared from wood blocks decayed under laboratory conditions are shown in Figure 6. The Kruskal-Wallis H test was conducted to determine if termite feeding differed according to the drying regime. Mass loss by termite feeding was statistically different between groups at α = 0.05, (X^2^(3) = 11.821, *p* = 0.008).

### 3.5. Effect of N-Hexane Extract from Laboratory Decayed Wood on Termite Feeding

The paper disks immersed in decayed wood extract showed trends similar to those of field-decayed stakes. In the no-choice feeding test, mass loss caused by non-dried decayed wood extract was the lowest, followed by solvent and sound wood extract, but there were no significant differences between the treatments X^2^(2) = 4.622, *p* = 0.99.

In the case of termite mortality, the highest difference was displayed by starvation followed by the non-dried decayed wood extract, sound wood extract, and solvent. Mortality between the groups was significantly different, X^2^(3) = 9.497, *p* = 0.023 (Figure 7).

## 4. Discussion

### 4.1. Feeding Tests of the Stakes

In the preliminary observation (shown in Figure 2), termite aggregations were observed around the specimens prepared from the decayed part of the field stake during the first three days of exposure. However, by the fourth day, the aggregation shifted to sound specimens. According to Matthews and Matthews [10], many insects that are deprived of food will sample items they would otherwise reject. The initial aggregation might suggest the attempt to sample the decayed stakes, which later they found as unsuitable. *Reticulitermes speratus* mainly feed on dead wood and grass [11] and the refusal of decayed wood as a food source indicates alteration by *F. radiculosa* has caused the wood to become unappealing for consumption.

**Figure 3:**
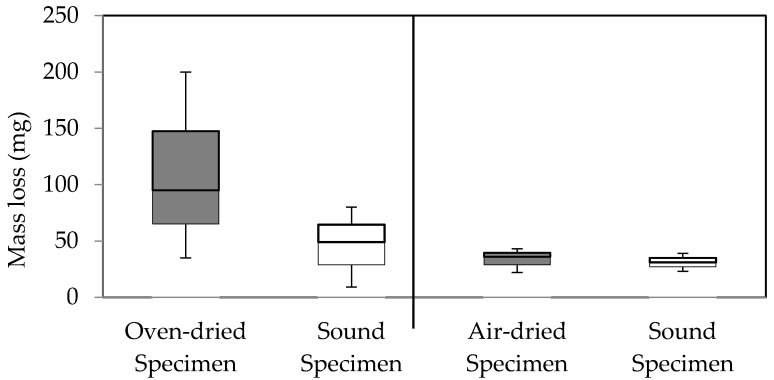
Comparison of the mass loss of oven- and air-dried specimens prepared from decayed stakes after termite feeding in the two-choice feeding tests. Sound specimens prepared from the sound part of the stakes (**open boxes**).

**Figure 4:**
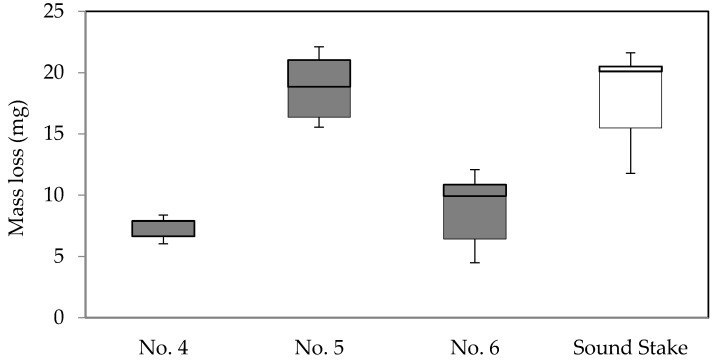
Box plot of mass loss of non-dried decayed stakes (**closed boxes**) in comparison with sound stakes (**open box**) after termite feeding in the no-choice feeding test.

Wood rot fungi may contain or release substances that signal the wood as unpalatable or heavily degraded to termites, leading to feeding deterrence. Insects are able use fungal secondary metabolites, as a signal of an unpalatable substrate, regardless of the original purpose for the production of such compounds [4,5]. Presumably, these cues can be eliminated by several factors. In Figure 3, differences were not detected between dried and sound specimens; this suggests that drying may reduce the feeding deterrent. External factors such as contaminants or degree of decay could affect the amount or availability of feeding deterrent. Amburgey and Smythe [12] presumed other organisms present in non-sterilized soil used for feeding tests altered termite feeding in wood decayed by *G. trabeum* isolate Madison 5096-15. Less termite feeding was recorded in decayed stakes No. 4 and 6 compared to stake No. 5, although the stakes had been exposed to soil for an identical period of time and originated from the same site (Figure 4). Decayed stake No. 5 was suspected of having a lower degree of decay or it could have been affected by other soil microorganisms.

### 4.2. Effects of the N-Hexane Extract from the Decayed Stake on Termite Feeding

**Figure 5:**
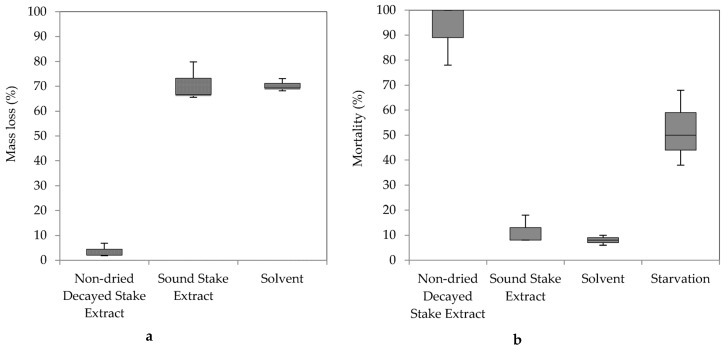
Box plot of mass loss percentage (**a**) and termite mortality (**b**) of paper disks immersed in the *n*-hexane extract of the non-dried decayed stakes and the sound stake in the no-choice feeding tests.

**Figure 6:**
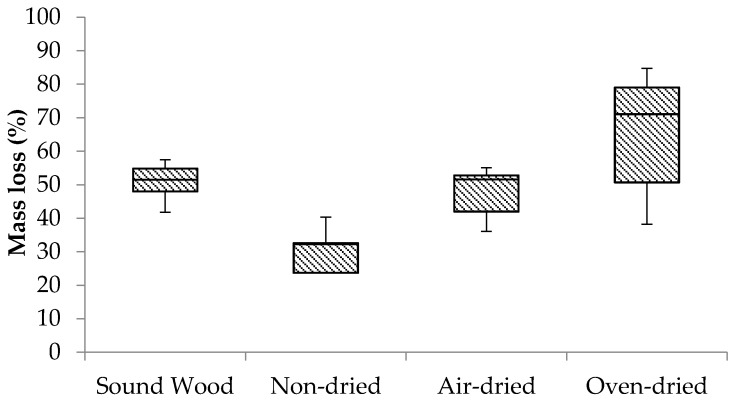
Box plot of mass loss (%) of wood specimens prepared from wood blocks decayed under laboratory condition in the no-choice feeding test.

**Figure 7:**
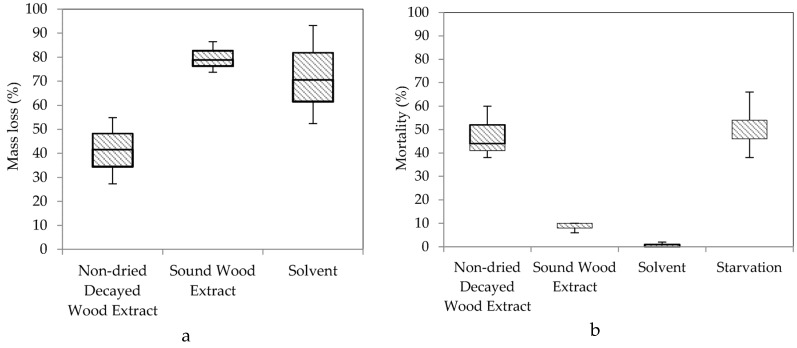
Box plot of mass loss percentage (**a**) of paper disks immersed in the *n*-hexane extracts of non-dried laboratory-decayed and sound wood and the mortality of termites (b) after 10 days exposure in the no-choice feeding tests.

A solvent-based extraction from fungal material could provide a solution containing a termite feeding deterrent compound(s) [7]. Decayed stakes were extracted using *n*-hexane and tested to observe their effect on termite feeding and mortality (Figure 5). Lower mass loss of paper disks immersed in the non-dried decayed stake extract compared to the sound stake extract and solvent demonstrated a termite feeding deterrent was in the *n*-hexane extract. Significant differences between groups was not detected despite the clear difference in median percent mass loss; this may be the result of generalization by Kruskal-Wallis H tests due to two medians (sound stake extract and solvent) close to one another (Figure 6). Notable termite mortality and low mass loss on paper disks treated with non-dried decayed stake extract suggests toxicity (Figure 6). Termites may have died because of poisoning instead of food rejection, because the number of termites that survived without any food was higher than those fed with non-dried decayed wood extract (Figure 7).

### 4.3. Feeding Test of Specimens Decayed by F. radiculosa under Laboratory Conditions

The result of the no-choice feeding test using decayed stakes indicated that termites avoided two of the decayed stakes (Figure 4). However, those stakes collected from the field also may have contained soil particles harboring a number of biological agents. To observe feeding deterrence without external factors we also conducted feeding tests using wood decayed by a pure culture of *F. radiculosa*.

Termite feeding was relatively low in non-dried samples, confirming the deterrent effect of *F. radiculosa* was present without the aforementioned external factors (Figure 6). Consumption of the air-dried samples was less than the oven-dried samples, indicating that air-drying reduces, but still retains the feeding deterrent, while oven drying removed the deterrent completely.

### 4.4. Effect of N-Hexane Extract from Laboratory Decayed Wood on Termite Feeding

Paper disks immersed in the *n*-hexane extract from decayed wood had the same feeding as those treated with sound wood extract or solvent. The range in the mass loss of paper disks immersed in the non-dried decayed wood extract may be a sign of uneven distribution of the feeding deterrent in the paper disks. At high concentrations, chemicals (in the form of feeding deterrent) released by fungi may act as a cue to termites that a food source is highly degraded and therefore inadequate [5]. If the chemicals were released at a lower concentration, it may signal the beginning of the decay process, indicating the existence of a lightly decayed food source.

It was observed, during the no-choice test, that aggregations around the decayed wood extract paper disks increased after day four. The feeding deterrent may have evaporated after four days, and cues provided by those compound(s) were no longer present. The percent mortality in the decayed wood extract treatments was higher compared to the other treatments (Figure 7b). The lower mortality in the sound wood extract treatment clarifies that the mortality was likely associated with the decay treatment. Termites fed moderately on paper disks treated with non-dried decayed wood extract, but in a pairwise comparison, the mortality median was not different than the starved termite treatment (*p* = 0.820) (Figure 7b). This suggests that termites may have accumulated a toxic compound by continuously ingesting the extract-treated paper disks. Further research is needed to identify the active compounds involved in the mortality or deterrence of termite feeding.

## 5. Conclusions

A feeding deterrent phenomenon was observed on field stakes decayed by a brown rot fungus, *Fibroporia radiculosa,* toward the subterranean termite *Reticulitermes speratus*. The compound(s) responsible for the termite feeding deterrence was easily removed by air-drying. There was variation in the mass loss attributed to termite feeding between three non-dried decayed field stakes; factors such contamination or degree of decay may have caused those differences. The feeding deterrent was removed by *n*-hexane extraction and exposure to the extract caused termite mortality. *R. speratus* feeding was deterred, under laboratory conditions, on wood decayed by *F. radiculosa*. Non-dried decayed wood was less consumed compared with air-and oven-dried decayed wood, and the highest feeding was recorded on oven-dried decayed wood. This result indicates drying may promote removal of the feeding deterrent in decayed wood. Paper disks treated with *n*-hexane extract from *F. radiculosa* decayed wood induced *R. speratus* mortality, suggesting the extract also contains a compound that is lethal to termites.
